# Bilateral clavicle fractures of the medial third treated by inverted anatomic locking plates: a case report

**DOI:** 10.3389/fsurg.2025.1562213

**Published:** 2025-05-13

**Authors:** Jian Tong, Ming Zhang, Qing Yu

**Affiliations:** Department of Orthopaedics, Taizhou School of Clinical Medicine, The Affiliated Taizhou People’s Hospital of Nanjing Medical University, Nanjing Medical University, Taizhou, Jiangsu, China

**Keywords:** bilateral medial clavicle fracture, incision and internal fixation, case report, inverted anatomic locking plate, shoulder function

## Abstract

Bilateral medial third clavicle fractures are very rare, with only very few cases reported in the literature. The mechanism underlying this type of fracture is often complex, and so far, its treatment remains controversial. We report a case of bilateral medial third clavicle fractures after a traffic accident. X-ray showed dislocation of bilateral medial third clavicles. The patient was cured by surgery and instrument fixation. Both clavicles were fixed using an inverted anatomic locking plate. The patient had excellent shoulder function after 2 years of surgery. Clavicle stability is necessary for normal shoulder function, and surgical fixation is becoming a trend for displaced medial third clavicle fractures. In this study, we reported a patient with bilateral clavicle fractures of the medial third who was treated by surgery and achieved excellent shoulder movement. An inverted anatomic locking plate is an effective internal fixation material for treating this type of fracture.

## Introduction

Clavicle fracture is very common in clinical practice, accounting for nearly 4% of all human bone fractures and up to 44% of all fractures of the shoulder girdle (1). However, medial third clavicle fracture is rare, accounting for less than 2%–3% of all clavicle fractures (2, 3). They are associated with multisystem injury, high energy trauma, and death (3). Furthermore, bilateral medial clavicle fractures are even rarer, and so far, only a few cases have been reported in the literature, accounting for less than 0.5% of all clavicle fractures (4). Most of the previous studies have suggested that non-operative treatment has been advocated as the golden standard for medial clavicle fractures due to the lack of specifically designed instruments and complex anatomical structures (5–7). However, conservative treatment of displaced sternal end fracture is often unsatisfactory with symptomatic injury and a nearly 15% nonunion (8). As previous studies have indicated unsatisfactory shoulder function and a high non-union rate among displaced fractures, surgery is increasingly used for medial clavicle fractures and various surgical methods have been proposed in this regard (3, 6, 9). In this report, we present a case of bilateral medial clavicle fractures treated by operation. The patient's management and postoperative functional status were assessed.

## Case presentation

A 56-year-old male was immediately transferred to our hospital following a serious traffic accident. He was riding on an electric bicycle and had a frontal collision against a telephone pole. Because of this accident, he suffered from multiple injuries, including the left fourth rib bone cortex distorted, minor pneumothorax, right-side pleural effusion, and bilateral medial third clavicle fractures. No other complications were found.

**Figure 1:**
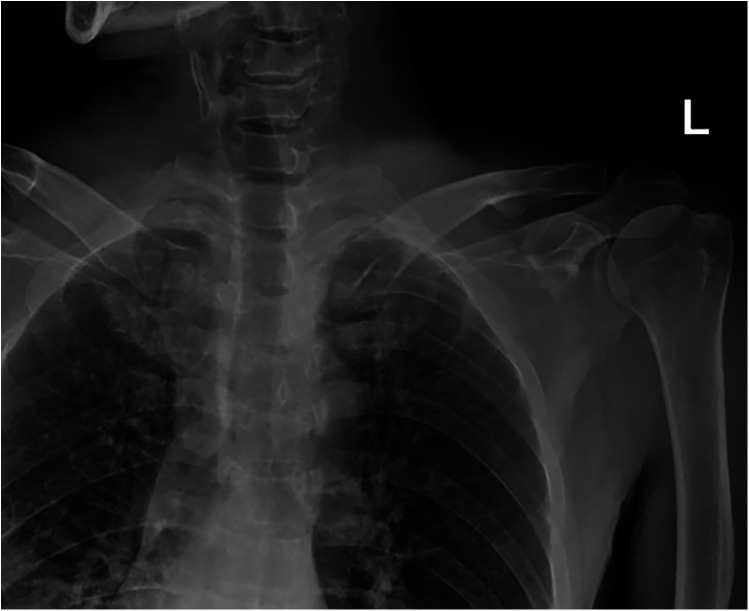
Anterior-posterior clavicle radiograph showing fresh bilateral fractures of medial clavicle.

**Figure 2:**
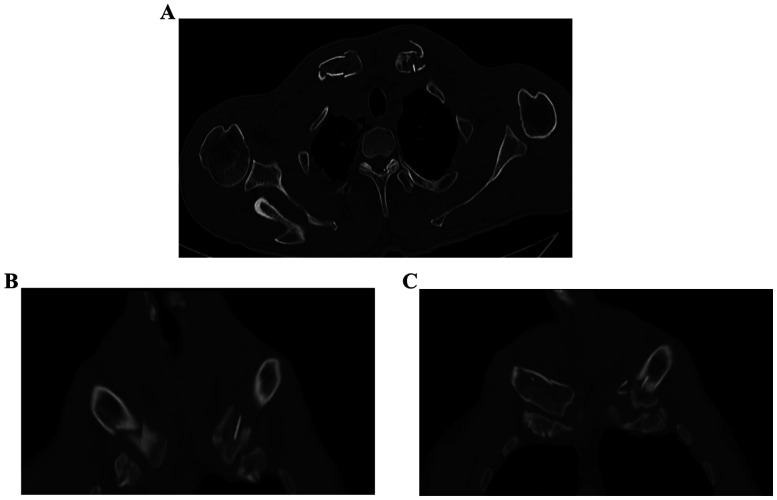
Ct image of bilateral medial clavicles showing complete displacement and shortening. **(A–C)** Are patient CT image of bilateral medial clavicles.

X-ray of the shoulder girdle exhibited a minimal displacement of both fractures (Figure 1). Considering his multiple injuries, conservative treatment was temporarily proposed. Both clavicles were immobilized by collar and cuff slings and the thorax was secured with a rib strap. After one week of observation, the patient still complained about serious pain around both clavicles and could not passively move the shoulder joint. A computed tomography (CT) scan was arranged to reveal the displacement and shortening of bilateral medial clavicle fractures (Figures 2A–C). The left clavicle fit type 1B2 and the right fit type 1B1 according to Edinburgh's classification. The examination also showed that pneumothorax and pleural effusion were stable and only needed conservative treatment.

**Figure 3:**
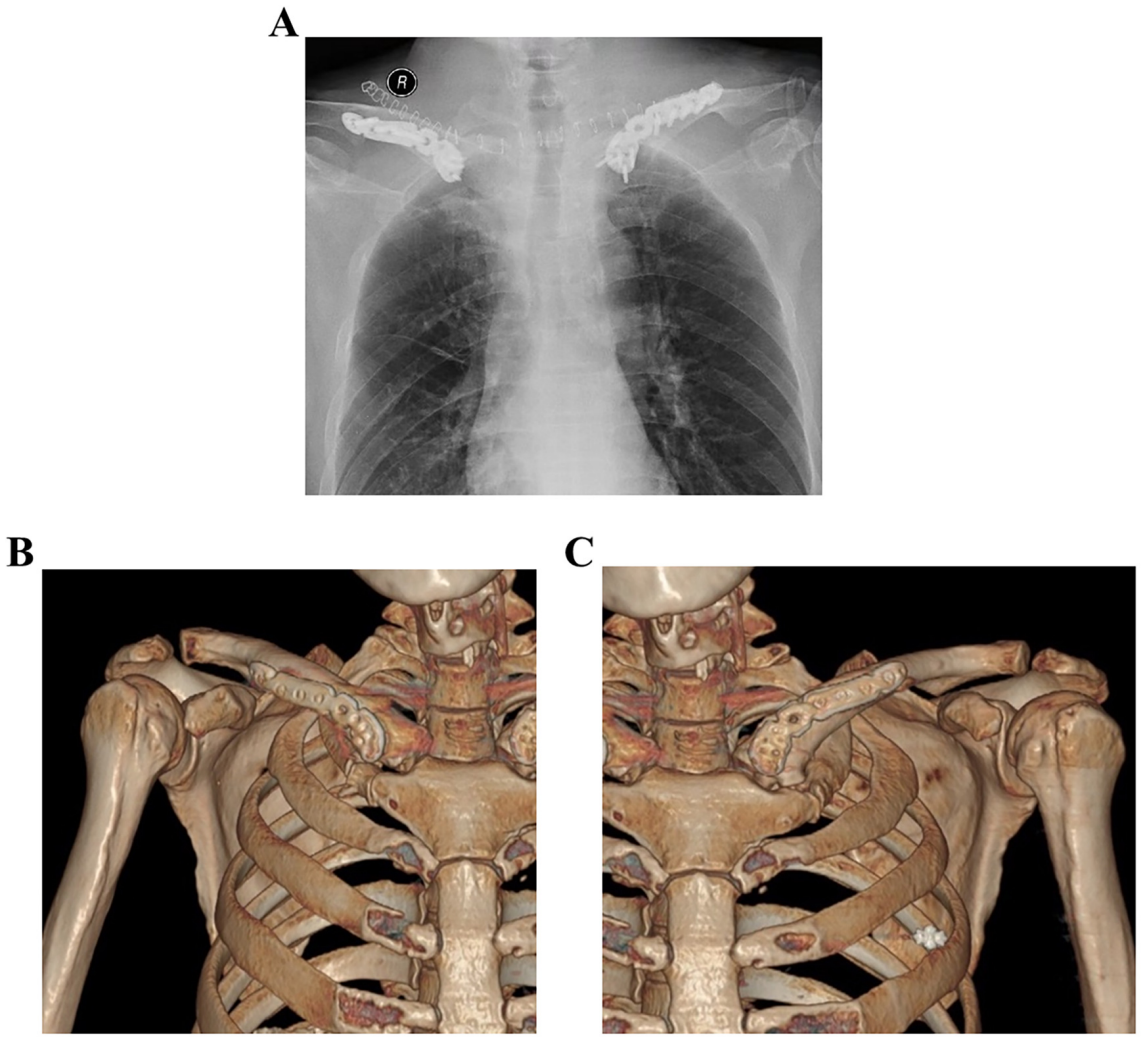
Postsurgical radiograph scans and CT scans. **(A)** Postsurgical radiograph demonstrating restoration of shoulder length after reduction and internal fixation with an inverted anatomic locking plate; **(B,C)** at eight months, CT scan revealed the union of both fractures.

**Figure 4:**
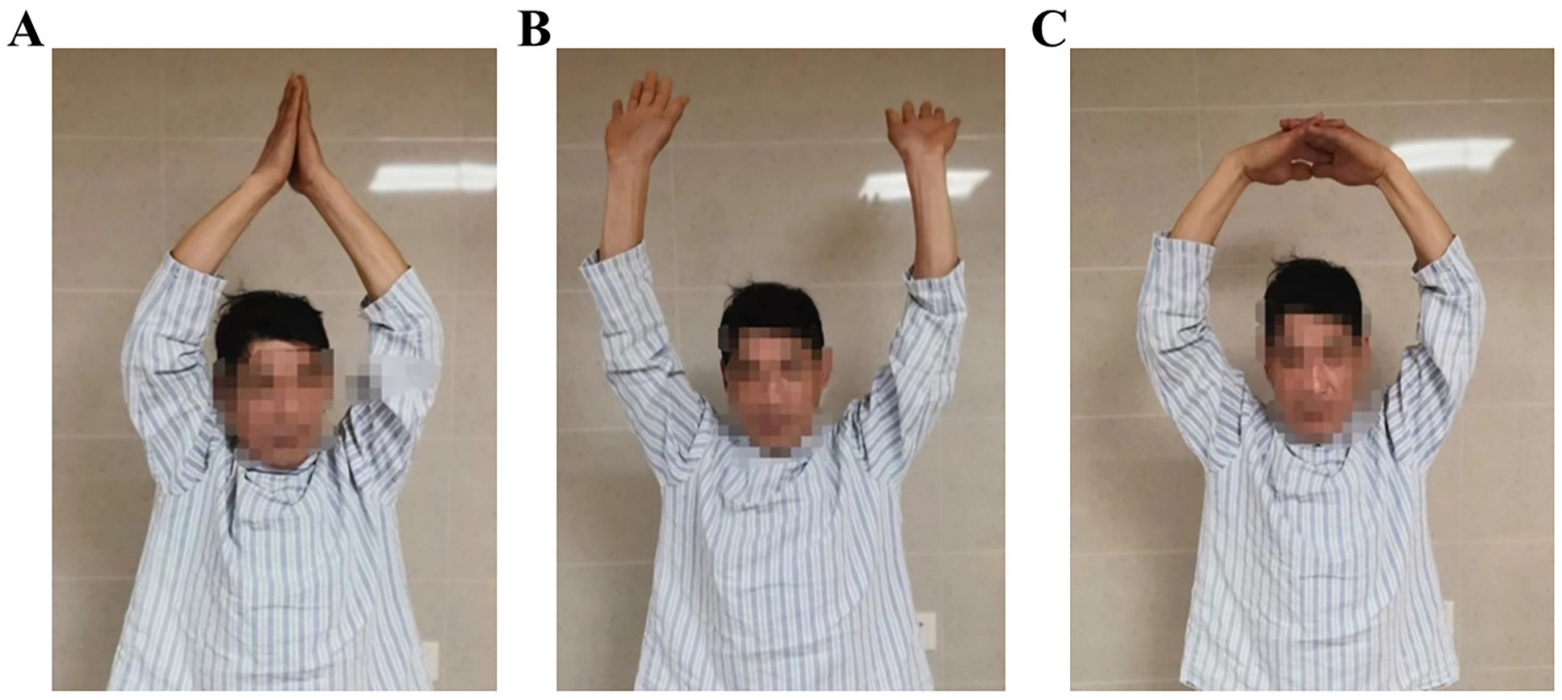
Free and full movement of the shoulder joints was achieved one year after the surgery. **(A–C)** Are patient activities.

Considering the displacement and intra-articular fracture, the patient finally underwent surgery and fixation with the instrument. Lateral locking clavicle plates (Double Medical, China) and reversed implantation were used to fix both clavicles. As the sternal end of the clavicle has a similar angulation and surface as the lateral, the inverted implantation is an ideal implant for facture fixation. The variable-angle screw holes located at the end of lateral locking plates were placed toward the medial clavicle; thus, screws could pass through and fix the medial-end fragments of the clavicle. The sternal end fragment was fixed by the 2.7 mm locking screw and the other side of the plate was fixed with the 3.5 mm locking screw. The surgery was conducted under general anesthesia and the shoulder was placed slightly high in the supine position. The fracture of the clavicle was exposed gently without periosteal damage. Then the medial fragment was fixed temporarily with Kirschner wire, and locking screws was used to fix the fracture. When plating, the fragment was fixed with locking screws that passed through the unilateral cortex because of adjacent major thoracic vessels such as the aorta and vena cava. Then, remove the temporarily fixed Kirschner wire and the surgical wound was carefully sutured. The same surgery was performed on both sides. The postoperative radiograph showed that the implants were in good positions (Figure 3A). After the surgery, both shoulders were allowed progressive functional exercise and active and passive movements of the shoulder started after 3 weeks. Two months after the surgery, the patient felt no pain during active movement. After six months, the patient had a good recovery and full range of motion. At eight months, CT scan revealed the union of both fractures (Figures 3B,C). The patient obtained the excellent function of the shoulder (Figures 4A–C) and returned to his daily activities. After two years of the surgery, we evaluated the shoulder function based on the Disabilities of the Arm, Shoulder, and hand (DASH) score. The result was 5.0 and there was no obvious hardware irritation. Informed consent was obtained from this patient and this study was approved by the TaiZhou People's Hospital Clinic Research Ethic Committee (KY 2022-113-01).

## Discussion

Bilateral clavicle fractures are rare. Most of the previously reported bilateral clavicle fractures were bilateral mid-range fractures or distal clavicle fractures (10–12). Bilateral medial clavicle fractures are even rarer, with only a few cases reported. Medial clavicle fractures are a special subgroup of clavicle fractures. The most common patients are middle-aged men experiencing traffic accidents. The high frequency of chest trauma (49%) and segmental fractures (9%) implies an association with high-energy trauma (3). The mechanism of this injury is complex including a motor vehicle or motorbike accident (36%) followed by sports (25%) and bicycle (23%) injuries (13). And it is usually associated with multi-organ and severe injuries and death (9, 14). This study reported a patient with bilateral fractures of the medial clavicle. The damage was mainly caused by strong violence directly affecting the chest.

Traditionally, non-surgical treatment is considered the gold standard for treating medical clavicle fractures. Undisplaced sternal end fractures warrant conservative treatment, and only few or no residual symptoms is occurred and the incidence of non-union for conservative treatment is less than 1% (15). Even in patients with significantly displaced fractures (6). However, the latest studies imply that conservative treatment of displaced medial clavicle fracture is often unsatisfactory. Displacement of sternal end clavicle fracture is most commonly defined in the literature using Throckmorton (>10 mm displacement) classification system (16). Following an average of three years non-operative management, the delayed union rate was 20% for displaced medial clavicle fracture (6, 17). Previous studies have found that 28% of patients who undergo non-surgical treatment may experience moderate-to-severe pain during follow-up (18). Robinson et al. showed that the non-union rate of non-displaced medial clavicle fracture is nearly 6.3% at 24 weeks after conservative treatment, while the proportion of displaced fractures is up to 14.3% (8). Salipas et al. found that only 45.8% of all patients undergoing non-surgical treatment achieve a good recovery. Of 7 patients with fracture displacement of more than 10 mm, 2 patients underwent surgery because of delayed union (6). Although, conservative management is still the major therapies, surgical treatment has recently been recommended for displaced medical clavicle fractures.

Surgical treatment of medial-end clavicle fracture is extremely challenging because of the complex anatomy and the limited options available for reliable internal fixation of the small medial fragment. Surgeries adjacent to the sternum can impose the risk of iatrogenic damage to the surrounding vasculature (19–21). As there is no special internal fixation for medial clavicle fracture, various implants have been used for fixation. There are three main ways of fragment fixation, including instrument plating (88%), suture (9%), and cerclage wires (3%) (2). Gill et al. used a modified hook plate and osteosynthesis, and the patient achieved a satisfactory shoulder function after a sixteen-month follow-up (22). A study found that displaced medial-end clavicle fracture can be fixed with an internal instrument with a union rate of 100% and a full range of motion at 12 months follow-up (23). However, implants have not been designed to fix the medial clavicle fracture. Most of the relevant studies are case reports, and there is no consensus on the optimal instrument for internal fixation. We used reversed lateral clavicle plates to fix medial clavicle fractures on both sides. The same surgical method was used in previous studies. Wang et al. published a patient with an extra-articular fracture of a medical clavicle who was treated with an inverted lateral clavicle locking plate. After 12 months of follow-up, the patient achieved satisfactory shoulder function and returned to daily activity (24). Bakir et al. used the same method to fix partial intra-articular fractures and achieved a good recovery (25). Liu et al. used the same method for 11 patients diagnosed with the medial-end clavicle, with no neurovascular injuries or internal fixation failures and good functional scores of the shoulder (26). It was found that fixation with lateral locking clavicle plates placed in reverse direction is a reliable and effective treatment for medial clavicle fractures.

However, we did not find any studies using inverted anatomic locking plates to fix bilateral medial clavicle fractures. We presented a patient with medial clavicle fractures on both sides. We followed the patient's postoperative functional status, and the subjective outcomes and satisfaction were measured.

In general, bilateral medial clavicle fractures are rare. Surgical treatment of displaced medial clavicle fractures using inverted plate has been reported as the optimal method to achieve rapid recovery with few complications. Therefore, the inverted anatomic locking plate can be used to cure this injury, even in patients with bilateral medial fractures. In the future, more studies should be conducted to validate our findings.

## Conclusion

Bilateral fractures of the medial clavicle are rare, with only very few cases reported in the literature. Our patient underwent surgery to insert an inverted anatomic locking plate, and the follow-up demonstrated complete fracture union and good functional recovery.

## Data Availability

The original contributions presented in the study are included in the article/Supplementary Material, further inquiries can be directed to the corresponding author.
